# The use of technology in postgraduate medical education within radiology: a scoping review

**DOI:** 10.1186/s43055-022-00763-7

**Published:** 2022-04-19

**Authors:** Sakina Akoob, Khalida Akbar, Jacqueline Van Wyk

**Affiliations:** 1 Faculty of Radiology, University of Kwa Zulu-Natal, Durban, 4041 South Africa; 2 Faculty of Management Sciences, Durban University of Technology, Durban, 4041 South Africa; 3 Faculty of Medicine, University of Kwa Zulu-Natal, Durban, 4041 South Africa

**Keywords:** Postgraduate, Education, Radiology, Computers, Artificial intelligence, Technology, Telemedicine

## Abstract

Postgraduate radiology training has traditionally followed didactic approaches; however, complex reasoning skills and critical thinking are essential in the field of radiology. Therefore, the shortages of radiologists in Africa have necessitated the need to review the use of technology in postgraduate education to improve efficient training and service. This scoping review was conducted to map the evidence on the role of technology in postgraduate radiology education and practice. A systematic scoping review search strategy was undertaken to review material published between January 2005 and August 2020 on the use of technology in radiology education. Data from the included studies were extracted and analyzed for emerging themes and presented in response to the research question. Seven articles described studies from the African continent. The most popular technological intervention was telemedicine, and several niche areas of technology implementation were identified (blended learning, flipped learning, digital teaching files). Furthermore, the most challenging aspects relating to technology use remain fiscal and credentialing constraints. Technology plays a role in postgraduate radiology education through networks, synchronous and asynchronous applications. It has the potential to increase support to doctoral students in the African context and alleviate some stressors associated with traditional, face-to-face didactic programs.

## Background

There currently exists a myriad of problems pertaining to radiology postgraduate medical education, not only in Africa but also worldwide. One of the key concerns for radiology training programs is access to annual budgetary provisions and secure legal backing. Not only are programs poorly funded, but curriculum review is also inconsistent [16]. There is also a shortage of trainers with some lack of commitment to do the trainings. Postgraduate education additionally occurs in an environment that prioritizes service provision rather than training. Resident doctors usually work between 80 and 168 h per week which results in the training programs been managed as a conduit for cheap workforce and assistants to the trainers/training centers, rather than an educational enterprise [16].

Over the years, teaching and training at postgraduate level in the discipline of radiology has typically been offered through didactic lectures and used a ‘spoon-feeding’ model. This practice has had a limiting effect as it does not afford learners an opportunity to act or think independently. The student has no other choice but to become a passive receiver of information during this ‘teacher-centered’ educational approach. This traditional learning approach relies heavily on lectures where content is directly dispensed to learners often through a PowerPoint format. These traditional lectures mostly do not include hands-on demonstrations or the use of technology which could greatly supplement, pique interest or enhance the learning experience. The lecturer, who is considered an expert in the field, speaks for most of, if not the entire time, often resulting in little to no discussion and participation from the audience [37, 42].

Numerous reasons exist for the continued use of the ‘spoon-feeding’ concept as a principal and popular teaching strategy in radiology education over the decades. Some of these reasons are, but not limited to: (a) an easier and more manageable method as minimal preparation is required for lectures as opposed to more complicated and technologically inclined teaching methods, (b) most teachers assume that students are comfortable with the lecture presentations, where they are told exactly what they need to know, (c) students have become accustomed to this form of training as it requires little effort on their part and assists in the preparation of examinations as lecture content often clearly teaches to the test/examination, (d) medical and scientific lecturers prefer ‘old-school’ techniques, as this are familiar to them and often find it difficult to deviate from the norm, and (e) financial or technologic limitations resulted in the reliance on the use of PowerPoint and other non-interactive methods of learning [1, 37]

The traditional methods of education can impede creativity and independent learning as it does not promote much student engagement [1]. During these lectures, learners are required to pay strict attention to the presentation and educator, and therefore, there is no mental time for lateral thinking [1, 37]. In most cases, students struggle to remember sufficient information from the presentations and the lack of interaction leaves them incapable of applying the new information to new situations that was not discussed in the lecture [37].

Radiology is a diverse field in comparison with other medical specialties. It relies greatly on visual learning and incorporates knowledge of diagnostics, information technology, clinical and nonclinical medicine and procedural expertise for intervention encompassing the entire body [37]. ‘Spoon-feeding’ as a teaching strategy in a discipline such as radiology is highly destructive. Radiologists are required to think ‘out of the box’ and integrate various pieces of information to meet new challenges and solve novel problems as they occur. In clinical practice, radiologic findings and results are seldom straightforward and material as studied from a textbook or lecture is rarely encountered in exactly the same format in reality. It is therefore imperative that radiology postgraduate students not only be trained to acquire expert knowledge but also learn useful techniques that will enable them to solve problems and think creatively [37].

In recent years, significant efforts have been made to improve radiology teaching and training during residency by incorporating newer innovative teaching skills and pedagogical techniques. With the advent of educational technology and the worldwide spread of computer networks (Internet), online teaching, learning and assessment tools such as e-portfolios have been popularized in many postgraduate radiology program [2]. Such technologies offer a platform to facilitate academia and practice skill development in radiology education and have hence become invaluable tools for interactivity, simulation, collaboration and even self-testing [35, 42]. Bari [8]found that the U.S. Department of Education had conducted research into higher education students using online learning. It was concluded that students utilizing online learning tools performed better when compared to those that just relied on face-to-face lecture courses [8]. This was attributed to the fact that computer-based teaching coupled with case studies is likely to improve students’ problem-solving ability.

## Review question

There is a dire need for radiologists in the African and public sector. Given the high rate of trauma and motor vehicle accidents as well as the burden of oncological patients and HIV, radiologists are involved in the immediate assessment of the patient and provide an important step in the immediate and short- and long-term planning of surgery and medical treatment of the patient. Given the shortages of radiologists, this review would assist both in increasing the number of radiologists trained even with a limitation of consultants (teachers) and assist in finding alternative methods to ensure that the training time in radiology be used effectively.

Therefore, this review sought to scope the role of technology in the education of postgraduate radiology student.

## Objectives

The objectives were to map the evidence on the use and integration of technology in the postgraduate education with the view to inform postgraduate training of registrars in radiology. The objectives were:

- to investigate the effect and extent to which technology is being used for training and education in radiology.
- to provide recommendations on the use of technology in postgraduate education in radiology

## Methods

The scoping review was conducted the highlight the role that technology plays in postgraduate medical education in radiology. A scoping review is a research methodology tool for mapping areas of research. By providing an overview of extant literature over a specific time period, the scoping review assisted researchers and readers to identify gaps more easily.

## Steps of scoping review

Two reviewers collected journal articles that aligned with the research objectives and that were published from January 2005 to August 2020. The scoping review methods suggested by the Joanna Briggs Institute were strictly followed. These entail the three main steps for a successful scoping review pertain to identifying the relevant literature, selecting the most appropriate literature, and extracting and charting the data. These steps are outlined in the subsections that follow [17].

### Identification of relevant literature

International and local electronic databases were used to identify the literature. These databases included EBSCOhost, Google Scholar, SAJR, PubMed/MEDLINE, Emerald Insights, Cochrane Library, Science Direct and I Catalogue. The search strategy included the following keywords: "postgraduate AND radiology AND technology AND education."

### Selection of literature

The selection of appropriate literature was subject to screening against very specific inclusion criteria. The population of the study was determined to be postgraduate radiology medical students. Table 1 illustrates the specific inclusion and exclusion criteria that were adhered to [4].

**Table 1:** Illustrates the specific inclusion and exclusion criteria that were adhered to

Inclusion criteria	Exclusion criteria
January 2005 to August 2020 to date	Older than 2005
Must be available in English	Not aligned with PCC
Be available in full text	Not available in full text
Pertain to postgraduate radiology education and training	A systematic or scoping review
*Population:* postgraduate radiology medical students	
*Concept:* Use of technology to guide postgraduate education in radiology	
*Context:* Socio-economic considerations of medical education in lower-to-middle income African countries	

### Data extraction and charting of data

**Fig. 1:**
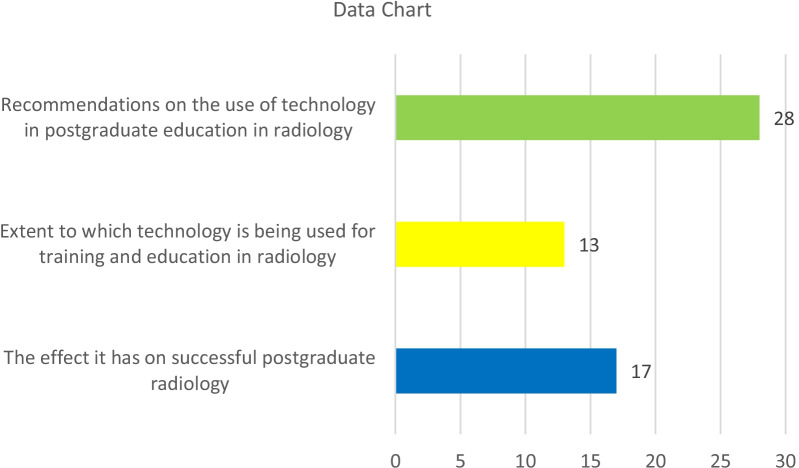
Is a data chart that was then created to summarise the data analysis and is presented in this figure

All the relevant literature was captured initially into EndNote and Microsoft Excel. The data were then screened against the specific inclusion and exclusion criteria presented in Table 1. A data chart was then created to summarize the data analysis and is presented in Fig. 1 [45].

## Results

**Fig. 2:**
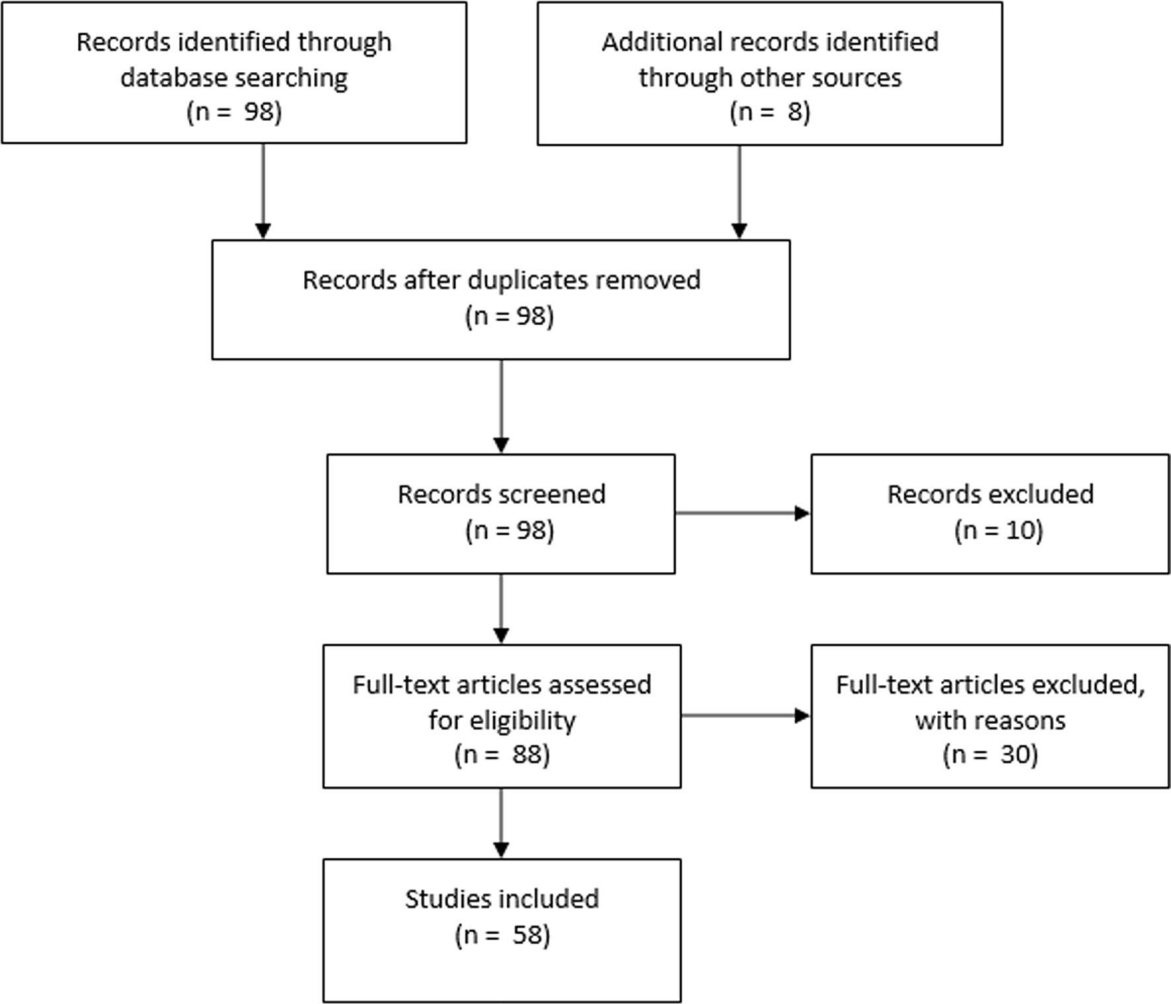
Highlights the comprehensive screening approach that was followed

The initial searches across the electronic databases yielded 1800 citations. The next step entailed the screening of titles and abstracts for eligibility, and 98 articles were identified. The next round of screening removed duplicates, scoping reviews and articles not aligned to the research objectives. This left a final subset of 58 publications that were included in the qualitative thematic data analysis. Figure 2 highlights the comprehensive screening approach as followed [26].

### Bibliometrics

**Fig. 3:**
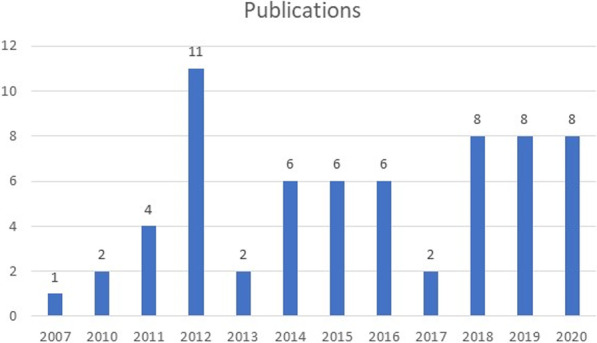
Presents the breakdown of publications across the years

The majority of articles included for review were published post 2010. There was a surge in publications in 2012 and thereafter a steady decline up until 2017. In the years from 2018 onwards, there has been a consistent stream of publications in this area. Figure 3 presents the breakdown of publications across the years, and Table 2 illustrates a breakdown of the focal areas covered across the publications included in this scoping review [45].

**Table 2:** Illustrates a breakdown of the focal areas covered across the publications included in this scoping review

Objective	Focus areas
Recommendations on the use of technology in postgraduate education in radiology	Address Challenges, Competency-Based Medical Education, Postgraduate Medical Education Evaluation, Address Covid-19 Challenges, Address Change, Decrease Case Loads And Increase Education, Web-Based Evaluation Forms, Web-Based Learning, Community Learning, Educational Videos, Pursuing Postgraduate Education In Radiology, E-Learning, Class/Conference Room Sessions, Social Media Usage, Assessing Professionalism, Artificial Intelligence, Web-Based Teaching, Emerging Technologies Adoption, Blended Learning
Extent to which technology is being used for training and education in radiology	Patient Education, Flipped Learning, Telemedicine, E-Learning, iPad Use, Blended Online Techniques, Technology-Enhanced Learning (Tel), Artificial Intelligence
The effect it has on successful postgraduate radiology	Gaming In Radiology Education, M-Learning, Mobile Technology, Social Media Profiles, Social Media, Raded, Adaptive Tutorials, E-Learning, Social Media, Case Comparisons, Digital Teaching Files, Simulation-Based Training, Digital Professionalism, Artificial Intelligence, Scientific Mobility

### Influence of technology on successful postgraduate radiology education

Several technologies were reportedly successful when implemented in postgraduate medical education for radiology students. Telemedicine [9, 14, 23, 38] has been found to be effective in supporting isolated healthcare professionals by providing them with the right expertise at the right time using affordable, low‐bandwidth technologies. Likewise, technology-enhanced learning (TEL) [10, 30] frees up time of students at educators because it combines face-to-face lectures and e-learning modules at their best convenience. Furthermore, e-learning projects facilitated the participation of learners enrolled in post-graduate programs across different regions that allowed for cross cultural sharing and the exchange of teaching experiences [5, 11].

This was found to be the case in Malawi where students valued the structured presentation of the basic sciences integrated into interactive virtual patients the access to e-journals and the opportunity for discussion with international surgical colleagues [43]. In addition, developing countries with critical shortages of healthcare workers benefitted from flipped learning, where medical students independently learn facts and concepts outside the classroom, and then participate in interactive classes to learn to apply these facts [31]. The implementation of flipped learning was useful as it engages learners by providing content before the live ("in class") session that aids in preparation and fosters greater in-class engagement [20].

### Application of technology being used for training and education in radiology

Technology was found to be highly effective when directly applied and used as part of training and education in radiology. For example, technological advances in gaming were more accessible in radiology residency programs [6]. Unlike more traditional videogames, games used in postgraduate medical education are inherently interactive, engaging and simulate complex decision-making processes [3, 47, 48]. Artificial intelligence [34] is the enabling technology at the forefront of these innovations; however, there are ethical concerns because the processes of medical device decision-making are largely unpredictable, therefore holding the creators accountable for it clearly raises concerns [22].

Likewise, the application of artificial intelligence can aid the training of radiologists regarding communication of diagnosis, consideration of patient’s values and preferences, medical judgment, quality assurance, education, policy-making and interventional procedures, because AI is able to identify findings either detectable or not by the human eye [33]. In a similar vein, social media technologies allow for the radiologists not to be restricted by geographic location and they are able to teach and learn asynchronously [12]. Social media can also form the bridge between the generational gap between seasoned radiologists who are educators and the new generation of radiologists to further enhance and enable effective training and education in radiology [21, 29, 44].

### Recommendations on the use of technology in postgraduate education in radiology

The majority of studies included in this scoping review provided a plethora of recommendations that assist in understanding the role of technology in postgraduate radiology education. Several challenges as identified in the literature are still hampering postgraduate education in radiology, which include fiscal constraints to the introduction of new technologies as well as accreditation and/or maintenance of competency [18, 28]. This creates a critical dilemma in the profession regarding credentialing because future radiologists need adequate training and expertise in conventional practice as well as new techniques [40, 49].

Furthermore, five factors were found that positively impact change in postgraduate medical education. These include shared commitment, reinvention, ownership, supportive structure and open culture [7]. On the other hand, factors that negatively impact change include resistance, behavior change, balance between different tasks, lack of involvement, lack of consensus, and unsafe culture and hierarchy [7, 13, 41]. Therefore, technological approaches to postgraduate medical education such as e-learning, web-based teaching, social media and artificial intelligence are necessary to assuage key intrinsic and extrinsic factors driving radiologists desire for personal professional development [24, 36, 39]. Key intrinsic factors included desire for personal professional development, desire for new challenges and search for satisfaction within the profession [19, 27]. Key extrinsic factors included requirements for continuous professional development, availability of funding, and search for improved remuneration [27, 46].

## Discussion

This study is the first to focus on the role of technology in postgraduate radiology education. The majority of publications included in this scoping review highlight the rapid pace of technological innovations that are augmenting traditional postgraduate teaching methods. These studies were published internationally, and the findings are applicable to the African context for postgraduate radiology training and education. The four most applicable technologies identified were e-learning, web-based teaching, social media and artificial intelligence, and South Africa does have the infrastructure to promulgate these approaches into the existing radiology resident programs. It is also likely that teaching under COVID-19 regulations had greatly led to development of suitable capabilities in the African context [15, 25, 32].

The limitations of this review were mostly due to the dearth of literature on postgraduate medical education for radiologists in the African context. This scoping review will contribute toward filling this gap, and future research can look into the effectiveness of implementing the four technologies identified as the most likely to have an impact on practice and future practices on the continent. A further limitation to this study was that almost all the publications reported on studies that were exploratory in nature. Future research can build on this scoping review to quantitatively evaluate the implementation of the range of technologies highlighted in this scoping review that can play a role in postgraduate education within radiology.

## Conclusions

The dilemma facing postgraduate radiology students and teachers is the lack of resources and practical limitations imposed by the scarcity of radiologists on the continent. This places a huge strain on practitioners who have to deliver services and train future radiologists under very challenging conditions. Therefore, the role of technology in postgraduate education is to assist radiology educators cope with these demands by reducing the time commitments and arduous experience of the current didactic traditional programs. Educators can engage and interact with students through avenues such as social media, e-learning, web-based teaching and teaching that incorporate artificial intelligence. Over time, these new technologies can be scaled up to provide wider coverage to more students that will impact on the steady increase in the number of radiologists.

## Data Availability

All data and material will be available to the journal upon request.
